# Role of DNA repair machinery and p53 in the testicular germ cell cancer: a review

**DOI:** 10.18632/oncotarget.13063

**Published:** 2016-11-03

**Authors:** Francesco Jacopo Romano, Sabrina Rossetti, Vincenza Conteduca, Giuseppe Schepisi, Carla Cavaliere, Rossella Di Franco, Elvira La Mantia, Luigi Castaldo, Flavia Nocerino, Gianluca Ametrano, Francesca Cappuccio, Gabriella Malzone, Micaela Montanari, Daniela Vanacore, Vincenzo Quagliariello, Raffaele Piscitelli, Maria Filomena Pepe, Massimiliano Berretta, Carmine D'Aniello, Sisto Perdonà, Paolo Muto, Gerardo Botti, Gennaro Ciliberto, Bianca Maria Veneziani, Francesco De Falco, Piera Maiolino, Michele Caraglia, Maurizio Montella, Ugo De Giorgi, Gaetano Facchini

**Affiliations:** ^1^ Progetto ONCONET2.0, Linea Progettuale 14 per L'implementazione della Prevenzione e Diagnosi Precoce del Tumore alla Prostata e Testicolo, Regione Campania, Italy; ^2^ Division of Medical Oncology, Department of Uro-Gynaecological Oncology, Istituto Nazionale Tumori ‘Fondazione G. Pascale', IRCCS, Naples, Italy; ^3^ Department of Medical Oncology, Istituto Scientifico Romagnolo per lo Studio e la Cura dei Tumori IRCCS, Meldola, Italy; ^4^ Department of Onco-Ematology Medical Oncology, S.G. Moscati Hospital of Taranto, Taranto, Italy; ^5^ Radiation Oncology, Istituto Nazionale per lo Studio e la Cura dei Tumori ‘Fondazione Giovanni Pascale', IRCCS, Napoli, Italy; ^6^ Pathology Unit, Istituto Nazionale Tumori “Fondazione G. Pascale”-IRCCS, Naples, Italy; ^7^ Department of Uro-Gynaecological Oncology, Division of Urology, Istituto Nazionale Tumori ‘Fondazione G. Pascale', IRCCS, Naples, Italy; ^8^ Epidemiology Unit, Istituto Nazionale per lo Studio e la Cura dei Tumori ‘Fondazione Giovanni Pascale', IRCCS, Napoli, Italy; ^9^ Psicology Unit, Istituto Nazionale per lo Studio e la Cura dei Tumori ‘Fondazione Giovanni Pascale', IRCCS, Napoli, Italy; ^10^ Department of Molecular Medicine and Medical Biotechnologies, University of Naples “Federico II”, Naples, Italy; ^11^ Pharmacy Unit, Istituto Nazionale Tumori, Istituto Nazionale Tumori-Fondazione G. Pascale Naples, Italy; ^12^ Department of Medical Oncology, CRO Aviano, National Cancer Institute, Aviano, Italy; ^13^ Division of Medical Oncology, A.O.R.N. dei COLLI “Ospedali Monaldi-Cotugno-CTO”, Napoli, Italy; ^14^ Scientific Directorate, Istituto Nazionale per lo Studio e la Cura dei Tumori ‘Fondazione Giovanni Pascale', IRCCS, Napoli, Italy; ^15^ Department of Biochemistry, Biophysics and General Pathology, Second University of Naples, Naples, Italy

**Keywords:** testis, germ cell cancer, DDR, ATM, p53

## Abstract

Notwithstanding the peculiar sensitivity to *cisplatin-based* treatment, resulting in a very high percentage of cures even in advanced stages of the disease, still we do not know the biological mechanisms that make Testicular Germ Cell Tumor (TGCT) “unique” in the oncology scene. p53 and MDM2 seem to play a pivotal role, according to several *in vitro* observations, but no correlation has been found between their mutational or expression status in tissue samples and patients clinical outcome. Furthermore, other players seem to be on stage: DNA Damage Repair Machinery (DDR) , especially Homologous Recombination (HR) proteins, above all Ataxia Telangiectasia Mutated (ATM), cooperates with p53 in response to DNA damage, activating apoptotic cascade and contributing to cell “fate”. Homologous Recombination deficiency has been assumed to be a Germ Cell Tumor characteristic underlying *platinum-sensitivity*, whereby Poly(ADP-ribose) polymerase (PARP), an enzyme involved in HR DNA repair, is an intriguing target: PARP inhibitors have already entered in clinical practice of other malignancies and trials are recruiting TGCT patients in order to validate their role in this disease. This paper aims to summarize evidence, trying to outline an overview of DDR implications not only in TGCT curability, but also in resistance to chemotherapy.

## INTRODUCTION

Testicular Germ Cell Tumor (TGCT) is a relatively rare neoplasm, affecting mostly young men between 15 and 40 years (incidence rate ≈ 1%): it is the most common type of cancer in this age range, with a peak incidence in the third and fourth decade of life. By considering histological features, TGCTs are usually categorized into two subgroups: seminoma and nonseminoma, the latter with an earlier peak incidence than the former (young adults aged between 25 and 29 years with a non-seminoma tumor diagnosis *versus* patients in the fourth decade of life affected by seminoma) . Although these two histological variants share the same risk factors, such as cryptorchidism and infertility, a statistical significant correlation between *in utero* environmental pollutants exposure and non-seminoma was highlighted [1, 2]. Probably due to differences in their progenitor cells, seminoma and non-seminoma disclose distinct clinical features and treatment strategies [3, 4], with a more aggressive biological behavior of non-seminoma.

In fact, seminoma has undoubtedly a better prognosis than the non-seminomatous counterpart, disclosing only intermediate and good risk subgroups, with no high risk sub-group unlike the non-seminoma[5].

Nevertheless, both subtypes of TGCTs are highly curable and their distinctive sensitivity to cisplatin-based therapy (and for seminomas to radiotherapy) has been studied for many years[6].

This sensitivity translates into an outstanding cure rate of nearly 80% for patients with advanced disease, but to date we do not have a clear knowledge about biological features underlying this exceptional sensitivity [7]. By answering the question about what are the reasons of TGCTs chemosensitivity, we could not only get information on the biological characteristics underlying intrinsic or acquired treatment-resistance ( even in view of the different histotypes - seminoma *versus* non-seminoma) but also collect evidence in order to develop new therapeutic strategies that can enhance chemosensitivity in other solid malignancies.

## p53 AND MDM2 : TWO SIDES OF THE SAME COIN

About half of human solid tumors carries p53 mutations, which are usually associated with cancer aggressiveness and poor outcome, but rarely occurring in TGCTs (1-5%) [8, 9] ; a distinctive element in TGCT, unlike other malignancies, is the lack of correlation between immunohistochemical p53 overexpression and mutation [10, 11], with high levels of wild-type p53 protein [12, 13] .

The role that this feature assumes in response to *cisplatin-based* therapies has not yet been clarified and remains still controversial. Gutekunst assigned a key role to p53 in the cisplatin-induced apoptosis of TGCT-derived cell lines, with a significant decrease in cisplatin-hypersensitivity by silencing p53, and a direct correlation between the absolute level of p53 protein upon cisplatin treatment and the extent of apoptosis[14]. The correlation between p53 and cyclins (especially cyclin B1) expression in TGCT was also investigated [15].

On the other hand, Burger et al. found no significant difference in sensitivity to cisplatin of p53 wild-type NTERA-2D1 cells compared to NCCIT cells (mutated p53), suggesting a lack of correlation between cisplatin-induced apoptosis and p53 status, which led to the conclusion that DNA-damage induced apoptosis might be p53-independent [16].

In accordance with this preclinical evidence, another study compared p53 expression in tissue samples of 17 cisplatin-responsive and 18 cisplatin- unresponsive TGCT patients, with a detection rate of 59% in platinum-responsive samples, compared with 83% of the non-responsive tumors; furthermore, although p*53* mutation was detected in only 1 of 17 TGCT patients who benefited from chemotherapy, *no p53* mutation was found in the 18 resistant TGCTs[17].

A combined gene sequencing and immunohistochemical analysis, performed on both seminomas and non seminomas [18], revealed low p53 protein expression in most samples, with low p53 expression occurring in seminomas and high expression mostly in non-seminomas. No p53 mutation was detected in these tumor samples. Interestingly, metastatic TGCTs also exhibited low p53 expression, even with a significant decrement of p53 protein detection in distant metastases compared to primary tumors. Authors concluded that there was no significant difference in p53 mutation or expression status between *chemotherapy-responders* and those who relapsed or died of TGCT.

Therefore, despite some preclinical evidence, neither hypothesis for which wild type p53 overexpression underlies the hypersensitivity of TGCT to *cisplatin-based* therapies, nor that for which p53 mutation is the main cause of *chemoresistance,* seem to be supported by a strong clinical validation.

MDM2 is the other side of the p53 “coin”: the principal function of MDM2 consists in down-regulating p53 activity, increasing its degradation in an ubiquitin-dependent manner[19]. High levels of MDM2 seem to be an intrinsic characteristic of embryonal carcinoma, and, regardless of therapeutical response, all embryonal carcinomas show a pronounced MDM2 protein expression, without gene amplification [20]: other MDM2 up-regulation mechanisms, as enhanced gene translation and translocation, have been suggested [21, 22]. An analysis of 81 TGCTs showed a strong MDM2 nuclear immunoreactivity in 34 (41.97%), with a statistical significantly higher staining in non-seminomas than in seminomas . MDM2 positive tumors were more frequent in patients who developed distant metastases than in disease-free patients, and MDM2 expression was significantly associated with tumors exhibiting a greater node involvement than early stages tumors (I and II/A) [23]. The inhibition of MDM2-p53 interaction appeared effective, *in vitro*, to activate the apoptotic cascade, also in platinum-resistant cells [24, 25] but there is no clinical evidence about its role *in vivo.*

Recently, it has been shown that p53/Mdm2 alterations are found only in platinum-extreme resistant TGCT patients [26], but, as noted above, most *non platinum-responders* TGCT are p53 wild-type, pointing out the concept that other pathways are involved in cisplatin-resistance. The study of these p53/MDM2 alterations could have a relevant clinical impact in the field of high-dose chemotherapy with hematopoietic progenitor cell support which is based on high-dose carboplatin and is used in cisplatin-resistant tumors [27-29]. These genetic alterations could contribute not only to better define criteria to use cisplatin-based regimens, but also to define criteria of sensitivity to high-dose carboplatin-based chemotherapy and then to select patients eligible for this kind of therapy [30, 31].

## DNA REPAIR MACHINERY IN THE TESTIS CANCER

Reduced DNA repair capacity was found to contribute to the hypersensitivity of testis tumor cells to cisplatin, compared to cisplatin-resistant *repair-proficient* bladder cell lines [32]. Cavallo et al. assessed proficiency of embryonal carcinoma (EC) cell lines in repairing cisplatin-induced DNA damage, showing a reduced repair ability: this reduced capacity correlated with reduced Homologous Recombination (HR). Because PARP inhibition proved to be a successful strategy in HR-defective tumors cells, they validated effectiveness of these drugs as monotherapy in EC cell lines; furthermore, they observed a synergistic interaction between PARP inhibitors and cisplatin, as the former reduce the cell proficiency to repair DNA damage caused by latter [33] . According to this feature, Gutekunst et al. observed an increased cisplatin-induced apoptosis by triple knockdown of ATM, ATR, and DNA-PK, although they considered that such silencing would have resulted in a reduced activation of p53 and consequently a lesser cell death extent than DDR proficient counterpart [14] .

In partial contrast with preclinical evidences highlighted by Cavallo, Bartkova et al [34] assessed HR proteins, such ATM, in EC tissue samples, detecting high levels of phosphorylated ATM, usually in 2-10% up to about 40% of tumor cells in the most positive case. Conversely, in seminomas was found a very low rate of positive stain cells, (11 of the 19 seminomas showed less than 1% of cells with a positive staining for phosphoATM). Similarly, phosphoATM was commonly undetectable in teratomas. They therefore proposed the idea, although speculative, that the unique biological features of TGCTs, such as *platinum-based* chemotherapy exceptional sensitivity, might be related to a less marked activation of the DNA Damage Repair (DDR) Machinery. Even in the most positive type of TGCT, the EC, there was a lower detection of phosphorylated DDR proteins, such as ATM, Chk2, and H2AX, than carcinomas [35-38].

The idea that emerges from these observations is that TGCTs, especially seminomas, “retain” characteristics inherited from their progenitor cells: spermatogones could be “programmed” to trigger the apoptotic process in response to minimum DNA damage, in order to prevent hazardous genetic mutations in the germ-line, and, therefore, in the progeny [39] . This feature of germ cells and TGCTs may underlie the exceptional curability of these tumors by DNA damaging agents, such as platinum-based chemotherapies or ionizing radiations, unlike other solid tumours.

A model of *cisplatin-induced* DNA damage resistance among TGCT is non-seminoma, especially embryonal carcinoma: their high constitutive DDR activation among all types of TGCTs [34] might explain resistance to DNA damage therapies. ECs, and other non-seminoma TGCTs, may experience a “cellular reprogramming”, with the expression of proteins, normally downregulated in germ cells, but often upregulated in carcinomas, that may contribute to the *platinum-resistant* phenotype .

*ATM single nucleotide variants* (SNV) were detected in 42% of TGCT samples, as well as the highest number of variants for a single gene - 21 (48% of all variants)[18]. The meaning of these SNV is still obscure: it might be interesting to study the activity of the proteins encoded by these genes to understand their role in the “economy” of the cancer cell, discriminating between “passengers” and “drivers” mutations.

Spermatocytic seminoma is a very rare variant of classic seminoma, accounting for 0, 61% of all germ cell tumors: intriguing features are the inability to metastasize, unless there are sarcomatous changes, and a favorable outcome with orchiectomy only [40]. Such characteristics make this tumor even more curable than classic seminoma, and comparison studies could be very attractive, to elucidate the molecular basis underlying these differences.

## DNA REPAIR MACHINERY : A FEASIBLE THERAPEUTICAL TARGET ?

Oncogene activation induces DNA replication stress, formation of DNA single (SSBs) and double strand breaks (DSBs) and subsequent response of the DNA Damage Repair (DDR) machinery [41, 42], as well as DNA damaging agents, such as platinum-compounds, capable of inducing both SSBs and DSBs [43].

Homologous recombination is the “error-free” arm of DNA repair machinery by using sister cromatids as template strand [44], despite having the defect of invariably leading to loss of heterozygosity and translocations or other gene rearrangements [45].

To date we have not yet realized molecular basis of resistance or sensitivity of various cancers to different therapeutical agents: Helleday supposed that this difference is due to specific DNA repair defects, which may be overwhelming in a cancer subtype rather than another [46].

In this regard, ovarian cancers are mostly responsive to carboplatin-based therapy and such sensitivity can be explained by decreased expression of proteins involved in Homologous Recombination, like BRCA or FANCF [47, 48], or by their mutations [49, 50]. Conversely, acquired platinum-resistance may occur with re-expression of FANCF 47 or genetic reversion of BRCA1 or BRCA2 mutations [49-51], highlighting the central role of this pathway in resistance mechanisms, as well as in therapy response . Similarly, the HR protein RAD51 correlated with increased DNA damage repair induced by etoposide (a drug used in combination with cisplatin also in the treatment of TGCT) and resistance in small cell lung cancer cells [52, 53].

However, loss of one or few HR proteins doesn't affect tumor cell viability: if there is a mutation in a HR gene, other HR pathways may overcome this deficiency; this concept is known as *synthetic lethality*.

In 1922, Calvin Bridges described the phenomenon in *Drosophila Melanogaster* specimens, but the term *synthetic lethality* was coined by Theodore Dobzhansky*.*

The concept is resumable in the capacity of a cell to make less of a gene (or protein) through another alternative pathway: if the “subrogor” pathway is lost, for a mutation or a pharmacological inhibition, the cell dies. This phenomenon is due to the inclination of organisms to maintain multiple pathways, able to counterbalance each other despite environmental changes or random events, such as mutations, in order to mitigate their effect on the cellular economy.

In this perspective, an interesting combination assessment has been proposed by Westphal and colleagues: both *in vitro* and in a murine model, contemporary loss of ATM and p53 lead to an enhanced radiosensitization [54].

Other groups found similar evidence: ATM knockdown in p53-deficient mouse embryonic fibroblasts resulted in an increased susceptibility to topoisomerase I and II inhibitors and to antimetabolites drugs, but not to agents like platinum compounds, or mitotic fuse poisons, like taxanes. Interestingly, loss of ATM function resulted in an increased *non apoptotic* cell death, as evidenced by Trypan Blue staining, suggesting that cytotoxicity may be mediated by *non-apoptotic pathways* [55].

Nevertheless, Toulany et al. [56] investigated the radiosensitizing effect of cisplatin in Non-small cell lung cancer (NSCLC) cell lines and in human fibroblasts (ATM-deficient and ATM-proficient) .

They observed an overexpression of phosphorilated ATM in radio-resistant A549 NSCLC cells upon cisplatin treatment, with a significant radiosensitization when ATM was inhibited by KU-55933: furthermore, radiosensitivity of A549 cells was synergistically enhanced by KU-55933 and cisplatin combined treatment.

According to these results, ATM-deficient cells were more sensitive to ionizing radiation upon cisplatin than the ATM proficient counterpart. Interestingly, A549 NSCLC are p53 wild-type and MDM2-overexpressed cells: various evidence suggest that MDM2 inhibition could enhance radiosensitivity [57] or act as a chemosensitizing agent to etoposide [58] .

Combined assessment of ATM and p53 is useful to predict clinical response to DNA Damaging Agents [59], which display an outstanding effectiveness in p53-deficient settings when ATM is suppressed.

On the other hand, ATM-deficient cancer cells are strongly *nononcogene addicted* to DNA-PKcs for survival after DNA damage, to such an extent that DNA-PKcs inhibition sensitizes ATM-deficient tumors to genotoxic chemotherapy.

Genetic alterations developed by tumor cells during neoplastic progression play a dominant role in response to chemotherapy and in susceptibilities to therapies in human malignancies: authors conclude that this observation is consistent with a cell death mechanism *other than apoptosis* ( see above ), a so-called *mitotic catastrophe*, in which cells, progressing through the cell cycle despite the presence of damaged DNA, trigger a mitosis-specific death program, being unable to preserve genome integrity.

**Figure 1:**
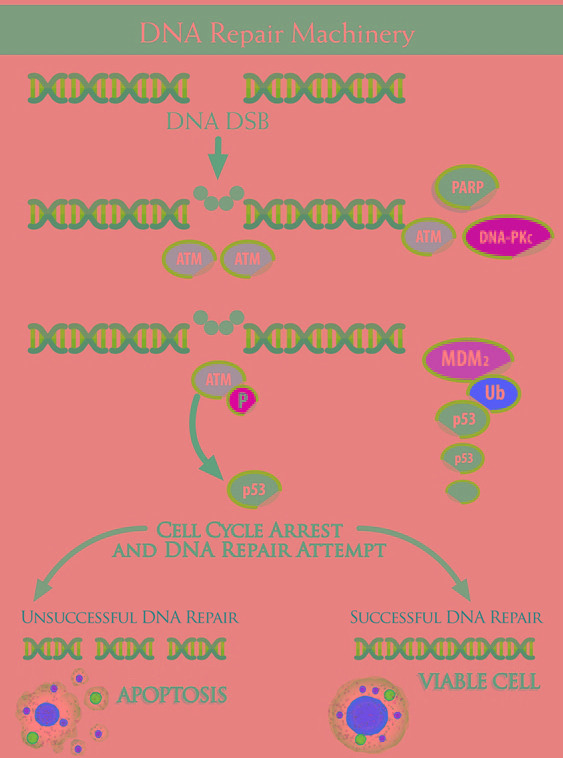
The machinery is composed of multiple “devices”, which cooperate from the detection of DNA damage to the cellular response ATM acts as protagonist, together with PARP, in the so-called “error-free” DNA repair Homologous Recombination - HR -, whereas DNA-PKc is the pivot in the “error-prone” Non Homologous End Joining - NHEJ. ATM ( Ataxia Telangiectasia Mutated) is present in form of inactive dimers or polymers within the cell: DNA Double Strands Breaks - DNA DSB - trigger ATM phosphorylation, with dimers dissociation: activated ATM monomers phosphorilate p53, which arrests cell cycle in expectation of DNA repair. With a successful repair, the cell remains viable, whereas , if the DNA Repair fails, p53 trigger the apoptotic cascade. p53 is down regulated by Mdm2, which lead to its degradation in an Ubiquitin-dependent process.

## THERAPEUTICAL PERSPECTIVES

As mutated p53 appears mostly an “undruggable” target, probably also due to *gain of function* mutations (GOF) [60, 61], whose biological meaning goes beyond the simple loss of DNA binding ability, many efforts have been made to target Mdm2 and several clinical trial are *ongoing* to validate safety and efficacy of drug inhibiting its activity (*ClinicalTrials.gov ID :NCT01877382 - NCT02098967 - NCT01677780*)

As seen above, PARP inhibition may be an intriguing issue: PARP1 was evaluated in 124 TGCT patients tumor specimens and overexpression was observed in Intratubular Germ Cell Carcinoma (100% of samples exhibited PARP1 overexpression), seminona (52.6%), EC (47.0%), yolk sac tumor (33.3%), teratoma (26.7%), and choriocarcinoma (25.0%), compared to 1.9% of normal testis specimens, showing no association between PARP1 expression and clinical variables [62]. This topic confirms the idea whereby DDR, and so PARP, is early activated in the development of TGCTs: subsequently, mutations in tumor genome can occur, with the loss of the PARP function, but, probably, with the hyperactivation of new “vicariant” pathways. A phase II trial of *olaparib* alone in patients with relapsed/refractory metastatic germ cell cancer is in progress (*ClinicalTrials.gov ID: NCT02533765*)

Furthermore, a combination study of *veliparib*, another PARP inhibitor, plus gemcitabine and carboplatin is recruiting patients with refractory TGCTs (*ClinicalTrials.gov ID: NCT02860819*).

In this regard, a phase I/II study of AZD0156, an ATM inhibitor, alone or in combination with olaparib, is recruiting patients suffering from various solid malignancies (*ClinicalTrials.gov Identifier : NCT02588105*), also in order to assess the efficacy of a *multiple HR protein inhibition* therapy.

In conclusion, we may assert that, exploring the wide landscape of DNA repair in human malignancies, we realized that broad tumor heterogeneity, even within the same tumor histotype, is now leading us towards an even more personalized medicine, and that only from the study of the molecular characteristics of each disease we can get the right information to give optimal therapeutic response.

### “At a glance”

- Testis Germ Cell Tumor (TGCT) is a neoplasia with “unique” biological and clinical behavior
- p53 and MDM2 are two sides of the same “coin” : their implications in TGCT sensitivity to DNA damaging therapies, as chemotherapy and radiotherapy, are still unclear
- DNA Repair Machinery (DDR) is an intriguing topic : Homologous Recombination (HR) Deficiency appears to be a feature underlying *cisplatin-sensitivity*
- In view of HR alterations, there is a biological rationale for the use of PARP inhibitors in TGCT
